# Host Genetics at the Intersection of Autoimmunity and COVID-19: A Potential Key for Heterogeneous COVID-19 Severity

**DOI:** 10.3389/fimmu.2020.586111

**Published:** 2020-12-22

**Authors:** Tugce Karaderi, Halin Bareke, Imge Kunter, Adil Seytanoglu, Ilgin Cagnan, Deniz Balci, Burc Barin, Mevhibe B. Hocaoglu, Nilufer Rahmioglu, Esra Asilmaz, Bahar Taneri

**Affiliations:** ^1^ Center for Health Data Science, Faculty of Medical and Health Sciences, University of Copenhagen, Copenhagen, Denmark; ^2^ Disease Systems Biology Program, Novo Nordisk Foundation Center for Protein Research, Faculty of Medical and Health Sciences, University of Copenhagen, Copenhagen, Denmark; ^3^ Department of Biological Sciences, Faculty of Arts and Sciences, Eastern Mediterranean University, Famagusta, Cyprus; ^4^ Department of Pharmaceutical Biotechnology, Faculty of Pharmacy, Institute of Health Sciences, Marmara University, Istanbul, Turkey; ^5^ Faculty of Pharmacy, Eastern Mediterranean University, Famagusta, Cyprus; ^6^ Vaccines and Infectious Diseases Therapeutic Research Area, The Emmes Company, Rockville, MD, United States; ^7^ Cicely Saunders Institute of Palliative Care, Policy & Rehabilitation, Florence Nightingale Faculty of Nursing, Midwifery & Palliative Care, King’s College London, London, United Kingdom; ^8^ Dr Fazil Kucuk Faculty of Medicine, Eastern Mediterranean University, Famagusta, Cyprus; ^9^ Wellcome Centre for Human Genetics, University of Oxford, Oxford, United Kingdom; ^10^ Nuffield Department of Women’s and Reproductive Health, University of Oxford, Oxford, United Kingdom; ^11^ Department of Gastroenterology, Homerton University Hospital, Clapton, United Kingdom; ^12^ Department of Genetics and Cell Biology, Faculty of Health, Medicine & Life Sciences, Institute for Public Health Genomics, Maastricht University, Maastricht, Netherlands

**Keywords:** COVID-19, SARS-CoV-2, immune response, autoimmunity, host genetics, susceptibility, cytokine, polymorphism

## Abstract

COVID-19 presentation is very heterogeneous across cases, and host factors are at the forefront for the variables affecting the disease manifestation. The immune system has emerged as a key determinant in shaping the outcome of SARS-CoV-2 infection. It is mainly the deleterious unconstrained immune response, rather than the virus itself, which leads to severe cases of COVID-19 and the associated mortality. Genetic susceptibility to dysregulated immune response is highly likely to be among the host factors for adverse disease outcome. Given that such genetic susceptibility has also been observed in autoimmune diseases (ADs), a number of critical questions remain unanswered; whether individuals with ADs have a significantly different risk for COVID-19–related complications compared to the general population, and whether studies on the genetics of ADs can shed some light on the host factors in COVID-19. In this perspective, we discuss the host genetic factors, which have been under investigation in association with COVID-19 severity. We touch upon the intricate link between autoimmunity and COVID-19 pathophysiology. We put forth a number of autoimmune susceptibility genes, which have the potential to be additional host genetic factors for modifying the severity of COVID-19 presentation. In summary, host genetics at the intersection of ADs and COVID-19 may serve as a source for understanding the heterogeneity of COVID-19 severity, and hence, potentially holds a key in achieving effective strategies in risk group identification, as well as effective treatments.

## Introduction

Coronaviruses have been a source of global alarm for the last two decades (1). The most recent emerging coronavirus SARS-CoV-2 has led to a pandemic, as declared by the World Health Organization (WHO) in March 2020. There have been more than 40 million confirmed cases of COVID-19 worldwide, and it has claimed more than 1,000,000 lives (2). Considering the risk of healthcare systems being overwhelmed, it is of utmost importance to pinpoint the risk factors for adverse disease outcomes to implement informed preventive measures limiting the global burden of the current pandemic.

Epidemiological reports from different countries highlighted COVID-19 mortality risk factors; comorbidities such as hypertension, diabetes, obesity, cardiovascular disease, chronic respiratory disease and cancer (3), as well as older age and male sex regardless of any comorbidities (2, 4). Discrepancies in testing availability, differences in testing algorithms and the presence of asymptomatic cases confound cross-country comparisons of the actual infection and mortality rates. These challenges are also relevant to research studies on host susceptibility factors for COVID-19 severity. Therefore, additional host susceptibility factors are likely to transpire as further epidemiological data emerge over time.

symptomatic and mild cases are estimated to comprise the majority of COVID-19 cases (5). As a multisystem disease, severe and critical cases may have acute respiratory distress syndrome (ARDS), and organ injuries due to cytokine storm and coagulopathies potentially leading to death (5–7). The striking interpersonal differences in clinical presentation have led to questions on the role of genetic factors. For instance, angiotensin-converting enzyme 2 (*ACE2)* was one of the first genes that attracted a lot of interest due to ACE2 being the viral portal of entry to the host cells, and the high mortality rate observed among cases with hypertension receiving ACE-inhibitor treatment.

Exaggerated inflammatory response is the culprit of the majority of the COVID-19 deaths. Hence, genetic susceptibility to dysregulated immune response is potentially among the host factors for adverse disease outcome. Such genetic susceptibility has also been observed in autoimmune diseases (ADs) (8, 9). Therefore, a significant question remains to be answered; does genetic susceptibility to ADs affect the risk for COVID-19–related complications and mortality?

ADs are complex diseases due to both genetic and environmental factors. They are characterized by an aberrant immune response to self-antigens due to the presence of autoreactive lymphocytes and loss of immune tolerance (10). Uncovering any potential genetic, and possibly, biological link between ADs and immune response to SARS-CoV-2 may in turn help to (1) identify individuals at risk, (2) shed light on COVID-19 immunopathology, (3) explain the broad heterogeneity in the disease progression and treatment responses, and (4) guide the vaccine development to prevent any vaccine-induced destructive immune response.

Herein, we briefly discuss the host genetic factors that have been under investigation with regards to association with COVID-19 disease severity, and also suggest a number of AD susceptibility genes, with the potential to be additional host genetic factors for heterogeneous COVID-19 presentation.

## Progress in the Investigation of COVID-19 Host Genetics

From the very start of the COVID-19 outbreak, protein members of the biological pathway essential for the entry of the virus into the host cells have become a focus of attention as candidate host susceptibility genes. Epidemiological findings on chronic conditions such as hypertension having more severe COVID-19 disease and a higher mortality risk, have also pointed out specific proteins functioning in the viral entry pathway (11). Of main interest were two proteins, ACE2 and transmembrane serine protease 2 (TMPRSS2), but the latter received significantly less attention with regards to host genetics studies (12–16). ACE2, a transmembrane protein, mainly found in airway ciliated epithelial cells with different enzymatic activities related to the renin-angiotensin-aldosterone system, has been shown to serve as a functional receptor for SARS-CoV-2 to infect nasal and alveolar epithelial cells in the lungs (17). The spike glycoprotein (S-protein) on the viral envelope of SARS-CoV-2 binds to the host ACE2 *via* its receptor-binding domain. Upon binding, the S-protein is activated by the TMPRSS2, which is a cellular protease that co-localizes with ACE2. This interaction assists the virus to fuse with the plasma membrane and facilitates the viral invasion of the host cell (18) (**Table 1**).

**Table 1:** Descriptions of selected host genes and their functions relevant to COVID-19 susceptibility and severity.

Gene	Location	Function	Discovery	Reference(s)
***CCR2***	3p21.31	The C-C Motif Chemokine Receptor 2 gene encodes the receptor for monocyte chemoattractant protein-1, a chemokine which mediates monocyte chemotaxis. *CCR2*-associated diseases include human immunodeficiency virus type 1 and idiopathic anterior uveitis.	GWAS/TWAS (differential predicted expression in lung tissue)	Pairo-Castineira et al. https://doi.org/10.1101/2020.09.24.20200048
***CCR3***	3p21.31	The C-C Motif Chemokine Receptor 3 gene encodes a receptor for C-C type chemokines. It belongs to family 1 of the G protein-coupled receptors. This receptor may contribute to the accumulation and activation of eosinophils and other inflammatory cells in the allergic airway. *CCR3*-associated diseases include aids dementia complex and folliculotropic mycosis fungoides.	GWAS/TWAS (differential predicted expression in lung tissue)	Pairo-Castineira et al. https://doi.org/10.1101/2020.09.24.20200048
***CCR9***	3p21.31	The C-C Motif Chemokine Receptor 9 gene encodes a member of the beta chemokine receptor family. Chemokines and their receptors are key regulators of the thymocytes migration and maturation in normal and inflammation conditions. *CCR9*-associated diseases include ileitis and celiac disease 1.	GWAS	PubMed ID: 32558485
***CXCR6***	3p21.31	The C-X-C Motif Chemokine Receptor 6 gene encodes a protein with G protein-coupled receptor activity and C-X-C chemokine receptor activity functioning in GPCR signaling and CCR5 pathway in macrophages. *CXCR6*-associated diseases include sarcoidosis 1 and immune deficiency disease.	GWAS/TWAS (differential predicted expression in lung tissue)	PubMed ID: 32558485; Pairo-Castineira et al. https://doi.org/10.1101/2020.09.24.20200048
***FYCO1***	3p21.31	The FYVE And Coiled-Coil Domain Autophagy Adaptor 1 gene encodes a protein with a role in microtubule plus end-directed transport of autophagic vesicles through interactions with the small GTPase Rab7, phosphatidylinositol-3-phosphate (PI3P) and the autophagosome marker LC3. *FYCO1*-associated diseases include autosomal recessive congenital cataract-2.	GWAS	PubMed ID: 32558485; Pairo-Castineira et al. https://doi.org/10.1101/2020.09.24.20200048
***LZTFL1***	3p21.31	The Leucine Zipper Transcription Factor Like 1 gene encodes an ubiquitously expressed protein localized in the cytoplasm. LZTFL1 regulates protein trafficking to the ciliary membrane via interactions with Bardet-Biedl Syndrome (BBS) proteins. It may also function as a tumor suppressor by interacting with E-cadherin and the actin cytoskeleton to regulate the transition of epithelial cells to mesenchymal cells. *LZTFL*-associated diseases include Bardet-Biedl Syndrome 17 and Bardet-Biedl Syndrome 1.	GWAS	PubMed ID: 32558485; Pairo-Castineira et al. https://doi.org/10.1101/2020.09.24.20200048
***SLC6A20***	3p21.31	The Solute Carrier Family 6 Member 20 gene encodes a membrane transporter with unidentified substrates within the Na+ and Cl- coupled transporter family. It is expressed in the kidneys. *SLC6A20*-associated diseases include iminoglycinuria and hyperglycinuria.	GWAS	PubMed ID: 32558485; Pairo-Castineira et al. https://doi.org/10.1101/2020.09.24.20200048
***XCR1***	3p21.31	The X-C Motif Chemokine Receptor 1 gene encodes a chemokine receptor belonging to the G protein-coupled receptor superfamily. It transduces a signal by increasing the intracellular calcium ions level. The viral macrophage inflammatory protein-II is an antagonist of this receptor. *XCR*-associated diseases include Leber plus disease.	GWAS	PubMed ID: 32558485
***NOTCH4***	6p21.32	The Notch receptor 4 gene encodes a member of the NOTCH family of proteins. Notch signaling is an evolutionarily conserved intercellular signaling pathway that regulates interactions between physically adjacent cells. This receptor may play a role in vascular, renal and hepatic development. *NOTCH4*-associated diseases include arteriovenous malformation and schizoaffective disorder.	GWAS	Pairo-Castineira et al. https://doi.org/10.1101/2020.09.24.20200048
***CCHCR1***	6p21.33	The Coiled-Coil Alpha-Helical Rod Protein 1 gene encodes a protein with five coiled-coil alpha-helical rod domains acting as a regulator of mRNA metabolism through its interaction with mRNA-decapping protein 4. *CCHCR1*-associated diseases include autoimmune psoriasis.	GWAS	Pairo-Castineira et al. https://doi.org/10.1101/2020.09.24.20200048
***HLA-G***	6p22.1	Human Leukocyte Antigen (HLA) G belongs to the HLA class I heavy chain paralogues on chromosome 6. HLA-G is expressed on fetal derived placental cells. *HLA-G*-associated diseases include asthma and severe pre-eclampsia.	GWAS	Pairo-Castineira et al. https://doi.org/10.1101/2020.09.24.20200048
***ABO blood group locus***	9q34.2	This locus encodes proteins related to the first discovered blood group system, ABO. Variations in the ABO gene determines the ABO blood groups. *ABO*-associated diseases include epiglottis cancer and orbital tenonitis.	GWAS	PubMed ID: 32558485; Replicated by the 23andMe (unpublished) and Zhao et al. https://doi.org/10.1101/2020.03.11.20031096
***OAS1, OAS2, OAS3 gene cluster***	12q24.13	The 2'-5'-Oligoadenylate Synthetase gene cluster encode the antiviral restriction enzyme activators, essential proteins involved in the innate immune response to viral infection. These enzymes are induced by interferons and catalyze reactions to activate RNase L, which results in viral RNA degradation and the inhibition of viral replication. This enzyme family plays a significant role in the inhibition of cellular protein synthesis, and hence, viral infection resistance. *OAS*-associated diseases include chikungunya, tick-borne encephalitis, microphthalmia with limb anomalies and pulmonary alveolar proteinosis with hypogammaglobulinemia.	GWAS/TWAS	Pairo-Castineira et al. https://doi.org/10.1101/2020.09.24.20200048
***TYK2***	19p13.2	The tyrosine kinase 2 gene encodes a member of the tyrosine kinase and the Janus kinase (JAK) protein families. It interacts with the cytoplasmic domain of type I and type II cytokine receptors and promote cytokine signals by phosphorylating receptor subunits. It is also a component of both the type I and type III interferon signaling pathways playing a potential role in anti-viral immunity. *TYK2*-associated diseases include hyperimmunoglobulin E syndrome (HIES) - a primary immunodeficiency characterized by elevated serum immunoglobulin E - and lymphomatoid papulosis as well as autoimmune diseases.	GWAS	Pairo-Castineira et al. https://doi.org/10.1101/2020.09.24.20200048
***DPP9***	19p13.3	The Dipeptidyl peptidase 9 gene encodes a protein that is a member of the S9B family in clan SC of the serine proteases. It has been shown to have post-proline dipeptidyl aminopeptidase activity. Dipeptidyl peptidases are involved in the regulation of the activity of their substrates and have been linked to a variety of diseases including type 2 diabetes, obesity and cancer. Other diseases associated with *DPP9* include pulmonary fibrosis and nasopharyngitis.	GWAS	Pairo-Castineira et al. https://doi.org/10.1101/2020.09.24.20200048
***APOE***	19q13.32	The Apolipoprotein E gene encodes an apoprotein of the chylomicron, which binds to a specific liver and peripheral cell receptor and is essential for the normal catabolism of triglyceride-rich lipoprotein constituents. Located on chromosome 19 in a cluster with the related apolipoprotein C1 and C2 genes. *APOE*-associated diseases include lipoprotein glomerulopathy, familial dysbetalipoproteinemia and type III hyperlipoproteinemia leading to increased blood triglyceride and cholesterol levels.	Observational candidate	PubMed ID: 32451547
***IFNAR2***	21q22.1	The Interferon Alpha And Beta Receptor Subunit 2 gene encodes a type I membrane protein that forms one of the two chains of a receptor for interferons alpha and beta. Binding and activation of the receptor stimulates Janus protein kinases, which in turn phosphorylate several proteins including STAT1 and STAT2. *IFNAR2*-associated diseases include immunodeficiency 45 and measles.	GWAS	Pairo-Castineira et al. https://doi.org/10.1101/2020.09.24.20200048
***TMPRSS2***	21q22.3	The Transmembrane Serine Protease 2 gene encodes a member of the serine protease family known to be involved in many physiological and pathological processes. It is upregulated by androgenic hormones in prostate cancer cells and downregulated in androgen-independent prostate cancer tissue. *TMPRSS2*-associated diseases include influenza.	Observational candidate	PubMed IDs: 32658335, 32664879, 32582302
***ACE2***	Xp22.2	The Angiotensin I Converting Enzyme 2 gene encodes a member of the angiotensin-converting enzyme family of dipeptidyl carboxydipeptidases. It has significant homology to human angiotensin 1 converting enzyme. It catalyzes the cleavage of angiotensin I into angiotensin 1-9, and angiotensin II into the vasodilator angiotensin 1-7. It also functions as a receptor for the spike glycoprotein of the human coronaviruses SARS and HCoV-NL63. *ACE2*-associated diseases include severe acute respiratory syndrome.	Observational candidate	PubMed IDs: 32341442, 32221983, 32658335, 32664879, 32582302

Literature search performed using the LitCovid hub for COVID-19 using the keywords ‘COVID-19 host genetics’ (https://www.ncbi.nlm.nih.gov/research/coronavirus/, access date 10 July 2020). GWAS, Genome-wide association study; TWAS, transcriptome-wide association study).

ACE inhibitors and angiotensin-receptor blockers (ARBs), which are used to balance blood pressure and vascular complications in chronic diseases (e.g. cardiovascular diseases and diabetes), provide various clinical benefits by increasing the expression of ACE2 while blocking ACE. Given that ACE2 facilitates viral invasion of human cells, there have been concerns that upregulation of ACE2, *via* use of ACE inhibitors and ARBs, may increase COVID-19 susceptibility and severity (19). However, it was also suggested that increasing ACE2 by the same intervention might be beneficial for a subset of cases, mainly because of its anti-inflammatory effects (19, 20). Supporting this perspective, a population-based case-control study reported that the use of ACE inhibitors or ARBs does not directly correlate with COVID-19 susceptibility or outcome severity (11). Studies in mice also suggested a potential protective role for ACE2 as its downregulation resulted in more severe respiratory failure (21). Furthermore, ACE2 deficiency has been shown to increase inflammatory response *via* increased expression of cytokines, promoting vascular inflammation (22). Hence, ACE2 may potentially play contrasting roles at different stages of the disease, affecting COVID-19 susceptibility and severity in multiple ways; at early stages, enabling viral entry to the cell, and hence, increasing disease susceptibility, and later, down-regulating cytokines/inflammatory response, and therefore, decreasing severity of the disease.

So far, studies on population genetics and genetic epidemiology of ACE2 variants have been inconclusive, not showing a significant global pattern. A number of potentially functional variants (such as missense variants rs758278442, rs759134032, and rs763395248) have been shown to have varying frequencies in populations of European vs. Asian descent, but convincing evidence for biological effects of these on disease susceptibility and severity require further functional experiments (23–27).

Another gene of interest has been apolipoprotein E (*APOE*), due to the observation of pre-existing dementia as a risk factor for COVID-19 severity and mortality in the older UK Biobank population (age range ~ 40–69 years; UKBB; **Table 1**). The UKBB study concluded that *ApoE* e4 allele increases the risk of severe COVID-19 infection, independent of pre-existing dementia, cardiovascular disease, and type-2 diabetes. Biologically, *ApoE* e4 plays a role both in lipoprotein function and in regulation of macrophage pro-/anti-inflammatory phenotypes (28). *ApoE* is highly expressed in the lungs (29). The precise biological mechanisms linking *ApoE* variants to COVID-19 severity require further investigation (30).

Further approaches to exploring COVID-19 host genetics involve international collaborations to perform hypothesis-free association analysis across the human genome. The global COVID-19 Host Genetics Initiative (HGI) has been formed to bring together international human genetics and epidemiology experts, and to gather and analyze scientific data on genetic determinants of COVID-19 susceptibility (31) (https://www.covid19hg.org/). Their main focus is to comprehensively investigate the human genome to obtain insights into disease susceptibility, as well as severity and outcome. As part of the Initiative, a genome-wide association study (GWAS) including 1980 cases from Italy and Spain was conducted by the Severe COVID-19 GWAS Group (32). Two loci, 3p21.31 gene cluster and 9q34.2 ABO blood group locus, were associated with severe COVID-19, defined by respiratory failure (**Table 1**). Individuals with the blood group A had a higher risk of severe COVID-19, whereas there was a protective effect for those with the blood group O. The gene cluster on chromosome 3 contains six genes (*CCR9, CXCR6, FYCO1, LZTFL1, SLC6A20, XCR1*) with potential roles in COVID-19 severity, such as those involved in immune response (*CCR9* and *CXCR6*), as well as in amino acid transport interacting with ACE2 (*SLC6A20*). A very recent GWAS preprint by Genetics of Mortality in Clinical Care (GenOMICC) collaborators including HGI (https://genomicc.org) reported additional novel loci (*DPP9*, *TYK2*, *OAS* gene cluster, *IFNAR2, CCR2, CCR3, HLA-G, CCHCR1*, and *NOTCH4*) associated with COVID-19 severity, all of which contain genes with roles in immune response and/or immune-mediate diseases (**Table 1**) (33). Although identifying actual causal gene variant(s) requires further association analyses in larger and more diverse sample populations as well as functional experiments, these results provide important insights into the potential factors affecting COVID-19 severity.

## Autoimmunity-Associated Genes Providing Potential Insights Into Susceptibility to Severe COVID-19

Immune system is key in shaping the outcome of SARS-CoV-2 infection. An appropriate immune response to SARS-CoV-2 is dependent not only on mounting the right type of response at the right time, but also at the right intensity. Exaggerated immune response to SARS-CoV-2, involving increased pro-inflammatory serum cytokines [e.g. interleukin-1B (IL-1B), IL-1RA, IL-7, IL-9, IFNγ, CXCL10, TNFα, and especially, IL-6 and IL-8], C-reactive protein, and lung inflammatory mononuclear infiltrates, may cause lung fibrosis and lead to life-threatening ARDS (34–37). Likewise, cytokine storm increases the risk for disseminated intravascular coagulation (DIC) and multiple organ failure that may result in death (38). Thus, it is mainly the deleterious unconstrained immune response, rather than the virus itself, that leads to COVID-19–associated mortality (39).

Several factors may cause the hyper-inflammation observed in severe COVID-19. One of them might be the weak early interferon response that leads to excessive viral replication, which then triggers an exaggerated inflammatory response (40, 41). Other factors promoting this aggressive immune response might be the initial dose of exposure and previous infections of the host. An important host factor is highly likely to be the host’s genetic predisposition to a dysregulated immune response, similar to those seen in ADs.

An intricate link is emerging between autoimmunity and COVID-19 pathophysiology. The presence of autoantibodies is associated with an increased need for respiratory support (42), and furthermore, anti-cardiolipin IgA antibody detected frequently in anti-phospholipid syndrome (APS) has also been observed in a number of COVID-19 cases, with thrombotic events (43, 44). Auto-antibodies against type I interferons have also recently been identified in some of the severe COVID-19 cases (45). Several groups have also put forth that the molecular mimicry by SARS-CoV-2 may induce a disseminated autoimmune reaction in the body (46–48). Interestingly, lymphopenia is both a prognostic factor in COVID-19 and a trigger in multiple ADs (35, 49–51).

Exacerbated immune response in COVID-19 is overall akin to hemophagocytic lymphohistiocytosis (HLH), with its hyperferritinemia, cytopenia, and increased cytokine levels (52). Interestingly, secondary HLH can be seen in rheumatological diseases, such as systemic lupus erythematosus (SLE), juvenile idiopathic arthritis, and rheumatoid arthritis (RA), which are all autoimmune conditions (53). Besides HLH, hyperferritinemia is a finding in a number of ADs such as adult-onset Still’s disease, and catastrophic APS (54). As per the observed link between autoimmunity and COVID-19, drugs commonly used in AD, such as corticosteroids, and IL-6R and IL-1 antagonists, are being tested and used in COVID-19 cases (55, 56).

Given the aforementioned link between autoimmunity and COVID-19, we propose a connection between genetic susceptibility to ADs and to COVID-19 severity. We put forth candidates for shared genetics, among severe COVID-19 and AD susceptibility, by considering the well-known and replicated genetic variants shared across multiple ADs (**Table 2**). As shown in **Table 2** and further discussed below, these genes code for proteins involved in antigen sensing, T cell activation and in cytokine signaling, hence they functionally participate in shaping the intensity of the immune response; a paramount factor in COVID-19 severity.

**Table 2:** Putative candidates for shared genes between severe COVID-19 and autoimmune disease (AD) susceptibility by considering the overlapping genetic associations across multiple ADs in genes that shape the immune response intensity.

Functional group	Gene	Location	Function	Associated autoimmune disease(s)	Reference(s)
**Antigen Sensing**					
	***TLR7***	Xp22.2	Toll-like Receptor 7; A pattern recognition receptor for single stranded RNA that is mainly expressed by plasmacytoid DCs (pDCs), B cells, and macrophages. Ligand binding induces the secretion of type 1 interferons, inflammatory cytokines and co-stimulatory molecules.	SLE	PubMed IDs: 19926489, 29374079, 32048277, 32706371
	***MHC***	6p.21	Major Histocompatibility Complex Class I; Expressed by all nucleated cells and is for intracellular antigen presentation to CD8+ T cells. Major Histocompatibility Complex Class II; Antigen presentation to CD4+ T cells by the antigen presenting cells (Dendritic cells, B cells and Macrophages)	Majority ot the ADs	PubMed IDs: 28449694, 20303870; Pairo-Castineira et al. https://doi.org/10.1101/2020.09.24.20200048
**Lymphocyte activation**					
	***PTPN22***	1p13.2	Protein tyrosine phosphatase, non-receptor type 22/ The lymphoid tyrosine phosphatese (lyp); lymphoid specific tyrosine phosphatase that is a negative regulator of T cell activation in T cell receptor signalling. It dephosphorylates Src kinases that are at the downstream of T cell receptor.	SLE, TID, RA, CD, TID	PubMed IDs: 22174698, 26502338, 19956096
**Cytokine Signaling**					
	***TYK2***	19p13.2	Tyrosine Kinase 2; Non-receptor type tyrosine kinase involved in signal transduction upon cytokine binding to its receptor. Receptors for IL-6, IL-12, Type I interferons, IL-23, and IL-10 use TYK2 for signal transduction.	RA, SLE, SS, JIA	PubMed IDs: 23143596, 26502338, 23603761, 26338038; Pairo-Castineira et al. https://doi.org/10.1101/2020.09.24.20200048
	***IL6R***	1q21.3	IL-6 receptor; A type 1 cytokine receptor that is mainly expressed on leukocytes. IL-6 is a pleiotropic cytokine that is involved in the acute phase response, neutrophil activation and Th17 differentiation.	AS, RA, SLE, JIA	PubMed IDs: 26974007, 24532676, 28714469, 23603761, 22491018

SLE, systemic lupus erythematosus; RA, rheumatoid arthritis; AS, ankylosing spondylitis; TID, type I diabetes; CD, Crohn’s disease; SS, systemic sclerosis; JIA, juvenile idiopathic arthritis.

In pathogen sensing by the innate immune system, toll-like receptor 7 (TLR7), an endosomal pattern recognition receptor (PRR), recognizes single-stranded RNA, and thus, it is among the initial innate immune cell receptors that sense SARS-CoV-2. TLR signaling leads to expression of pro-inflammatory cytokines and interferon genes (10). ADs, such as SLE, RA, and systemic sclerosis, have associations with TLR variants (10, 57, 58). Even though the early interferon response is delayed in SARS-CoV-2, upon increased viral load, excessive signaling due to genetic variants in TLR signal transduction pathways may lead to exacerbated macrophage and neutrophil activation, and subsequent, cytokine secretion (59). Interestingly, a recent case-series has shown that four young males with severe COVID-19 harbored rare *TLR7* variants, which hampered interferon responses upon TLR7 engagement (60). Since *TLR7* is located on the X-chromosome and is expressed bi-allelically, it has been suggested as one of the reasons for the sex bias, albeit in opposite direction, seen in AD and COVID-19 (61). These claims are yet to be proved, but it is becoming more apparent that a coordinated immune response is key for successfully controlling SARS-CoV-2 infection (62). Therefore, it is probable that any change in TLR7 signaling might increase the risk for a dysregulated immune response seen in severe COVID-19. The role of genetic variants in other players for type I interferon response, including *TLR3* in severe COVID-19 is also emerging (63). In the case of antigen sensing in the adaptive immune system, T cells are dependent on antigens presented on human leukocyte antigen (HLA) molecules, and HLA loci are strongly associated with ADs (64). However, the mechanistic link between HLA loci and autoimmunity is complex, hence it is challenging to put forth a specific candidate variant that might also be present in severe COVID-19 cases. The GWAS in Italian and Spanish populations did not find any link between the HLA locus and respiratory failure in COVID-19 (32). However, a recent small-scale Han Chinese study has shown that HLA-C*07:29 and B*15:27 genotypes were more common in 82 recovered COVID-19 cases (65). As a highly polymorphic region, any HLA association is also bound to be population-dependent, similar to the previously observed HLA associations with various ADs (66).

Upon antigen recognition, signal transduction through the T cell receptor (TCR) leads to the activation of T cells. Among the proteins involved in T cell activation, protein tyrosine phosphatase non-receptor type 22 (PTPN22) is a negative regulator of T cell signaling. *PTPN22^R620W^* variant is very frequently detected in a variety of ADs (**Table 2**) (67, 68). Interestingly, *PTPN22^R620W^* is a gain of function variant, where TCR signaling is more inhibited (69). Increased activity of PTPN22 might tip the balance in regulatory T (Treg) cell and effector T cell homeostasis against Tregs (70). Such a variant may also contribute to the T cell depletion and immunoparalysis seen in severe COVID-19 cases (51, 71).

Cytokines play a pleiotropic role in modulating leukocyte activity, differentiation and intensity of the immune response. Given the deleterious effects of hypercytokinemia in severe COVID-19 and association of IL-6 with disease severity, genetic variants that cause altered cytokine signaling might be among the host genetic factors for severe COVID-19 susceptibility (35). Janus kinase-signal transducer and activator of transcription (JAK-STAT) pathway is involved in signal transduction from cytokine receptors. Baricitinib, a selective JAK1/JAK2 inhibitor that is approved for RA treatment, is currently being tested for COVID-19 management (72). Tyrosine kinase 2 (TYK2) is a non-receptor tyrosine kinase from the JAK family that functions together with different JAK molecules, for signal transduction from IL6R, type I interferon receptors, IL-12, IL-23 and IL-10 receptors. *TYK2* variants have been shown to affect disease susceptibility in a variety of ADs (**Table 2**) (73, 74). Similarly, these *TYK2* variants may be associated with disease manifestation, as well as the response to Jak inhibitors (Jakinibs) in COVID-19. Response to Jak inhibitors is especially important as Jakinibs are among the prime drugs that are being tested for drug repurposing in COVID-19 (75) (clinical trials: NCT04320277, NCT04338958). Of great interest, during the peer review process of this perspective, the GenOMICC study identified *TYK2* association with critical illness in COVID-19 (**Table 1**) (33).

Furthermore, the *IL6R* gene variants have been associated with heterogeneous response to anti-IL6R (Tocilizumab) therapy in RA (76–78) (**Table 2**). Hence, in terms of inferring potential host genetic factors affecting treatment responses in COVID-19 from the genetic studies of ADs, the genetic variants of *IL6R* might potentially be promising biomarkers of response to tocilizumab in COVID-19 (78).

## Discussion and Future Directions

COVID-19 pandemic has had significant healthcare, socio-economic and personal implications, and led to extreme protective measures worldwide, such as lockdowns and border closures. Significantly increased mortality rates for the high-risk groups such as the elderly and those with chronic diseases, as well as concerns of overwhelmed healthcare systems, necessitated use of such drastic measures (3). Accumulating epidemiological data across countries have also been revealing potential new risk groups and post-infection effects of COVID-19 (e.g. acute thyroiditis in young individuals, an inflammatory condition similar to Kawasaki disease in children, and onset or worsening of diabetes (79–82)). There is a plethora of global research currently ongoing in this rapidly progressing field. Therefore, more factors and outcomes related to COVID-19 susceptibility and severity will become evident in the coming months.

Individuals with ADs and those predisposed to ADs (e.g. with family history for one or more ADs, and/or with clinically/genetically determined higher risk for ADs) are of special interest given the immune-related pathways and immunomodulatory treatments shared with COVID-19. In a number of epidemiological studies conducted, ADs have not been indicated as major risk-modulating co-morbidities for severe COVID-19 (83). However, new evidence from a large recent study from the United Kingdom has revealed RA, lupus and psoriasis, as risk factors for COVID-19–related deaths (3). Given the current contrasting epidemiological evidence, it should be considered whether a possible association might be masked by the use of some immunomodulatory drugs in ADs, or because underlying autoimmunity has not yet manifested (84). Investigating the family history of ADs and/or estimating the risk of individuals for having ADs, using available clinical and/or genetic variables in cases with severe and critical COVID-19, could be an approach to reveal a possible link between predisposition to ADs and COVID-19 severity. Even though consistent epidemiological connection between severe COVID-19 and AD is currently lacking, the shared biological pathways and genetic variants related to those pathways may still aid in deciphering the dysregulated immune response to the infection and identifying additional targets for treatment and drug repurposing.

Moreover, candidate gene studies and GWAS including larger samples of COVID-19 cases of diverse ethnic backgrounds may shed more light on the host genetic factors in severe COVID-19. Furthermore, performing sex-stratified genetic analyses in these larger sample populations is necessary for investigating the observed epidemiological sex difference in the COVID-19 susceptibility and severity. Although environmental/behavioral factors are likely to play a role, at least partially, in the observed sex differences, genetic and biological factors may also contribute to these observations (4). Sex differences observed in ADs and COVID-19 severity may also share a number of these factors.

Besides host genetic factors, it has been shown that cases with severe COVID-19 has a higher viral load and sustain this load for a longer period (85). Although it remains to be determined whether the high viral load is the direct cause of the severe disease, or it is due to a dysregulated immune response, viral load has emerged as a potential key player affecting COVID-19 severity. Additionally, mutations detected in the virus, especially in the spike protein, have a potential to alter its virulence (86). Overall, the current scientific evidence shows that COVID-19 severity is determined by a combination and interplay of host and viral factors. Thus, multidisciplinary and comprehensive studies are required to tackle the problem of severe COVID-19 and to unravel the biological mechanisms involved to reveal rational drug targets for better treatment.

The genetic underpinnings of ADs and the biological relevance of many autoimmune-associated variants are yet to be clearly elucidated. Thus, in this perspective, we focused on the immune-related genetic factors, frequently observed across different ADs, which may also be functionally relevant in COVID-19 severity and treatment. To contribute towards an effective response to the pandemic, we put forth a number of gene candidates such as *TLR7*, the MHC region, *PTPN22*, *TYK2* and *IL6R* (**Table 2**), albeit not exhaustive, that may affect the risk of having ADs, and modulate COVID-19 severity, as well as treatment response *via* common biological pathways. After preparation of this manuscript, supportive evidence for the role of the two of the gene candidates, *TLR7* and *TYK2*, in severe COVID-9 has already started to surface, underscoring the importance and relevance of using the knowledge gained on ADs to shed light on SARS-CoV-2 (33, 60).

## Data Availability Statement

Publicly available datasets were analyzed in this study. This data can be found here: Via literature search performed using the LitCovid hub for COVID-19 using the keywords ‘COVID-19 host genetics’ (https://www.ncbi.nlm.nih.gov/research/coronavirus/).

## Author Contributions

Performed the main literature search and wrote the first draft: TK, HB (contributed equally). Provided substantial literature search and writing: IK, AS, IC, DB. Critically evaluated and revised the manuscript: BB, MBH, NR, EA, BT. Conceptualization: TK, HB, EA, BT. Supervised the work: BT. All authors contributed to the article and approved the submitted version.

## Funding

TK was supported by the Novo Nordisk Foundation Center for Protein Research (grant NNF14CC0001). MBH was supported by the National Institute for Health Research (NIHR) Applied Research Collaboration South London (NIHR ARC South London) at King’s College Hospital NHS Foundation Trust. The views expressed are those of the author and not necessarily those of the NIHR or the Department of Health and Social Care.

## Conflict of Interest

BB was employed by The Emmes Company.

The authors declare that the research was conducted in the absence of any commercial or financial relationships that could be construed as a potential conflict of interest.
